# Association of surfactant protein D gene polymorphism with susceptibility to gestational diabetes mellitus: a case–control study

**DOI:** 10.1186/s12884-022-04541-1

**Published:** 2022-03-22

**Authors:** Jingwei Xu, Yi Chen, Liangfang Tang, Xinyuan Teng, Lin Feng, Ligui Jin, Guirong Wang, Liquan Wang

**Affiliations:** 1 School of Medicine, The Second Affiliated Hospital, Zhejiang University, Hangzhou, 310009 Zhejiang China; 2 The First People’s Hospital of Wenling, Wenling, 317500 Zhejiang China; 3 Department of Surgery, SUNY Upstate Medical University, UH Room 8715, 750 E Adams St, Syracuse, NY 13210 USA

**Keywords:** Allele, Gestational diabetes mellitus (GDM), Polymorphisms, SP-D

## Abstract

**Background:**

Surfactant protein D (SP-D) is a critical component of the innate immune system intrinsically linked to energy metabolism. However, the relationship of SP-D gene polymorphisms and gestational diabetes mellitus (GDM) remains unclear. In this study, we analyzed SP-D gene polymorphisms in GDM patients and nondiabetic controls and then determined the association of SP-D gene polymorphisms with GDM.

**Methods:**

We examined a common genetic polymorphism located in the SP-D coding region (rs721917, Met31Thr) in GDM patients (*n* = 147) and healthy pregnant controls (*n* = 97) by using a cleaved amplification polymorphism sequence-tagged sites (PCR–RFLP) technique. The level of SP-D protein in the serum of GDM patients and nondiabetic controls was determined by ELISA. The gene and allele frequencies of SP-D and their association with GDM as well as SP-D protein levels were analyzed and expressed as odds ratios (ORs) with 95% confidence intervals (95% CIs).

**Results:**

We found that there was a significant association of the SP-D polymorphism (rs721917) with GDM. The SP-D (T/T) genotype was found in 11.6% and 21.6% of GDM patients and matched healthy controls, respectively (odds ratio, 0.473; 95% confidence interval, 0.235–0.952; *P* = 0.033), indicating that women with the (T/T) genotype had a lower prevalence of GDM (OR = 0.473). Women with the T/C genotype showed an increased risk of GDM (odds ratio, 2.440; 95% confidence interval, 1.162–5.123; *P* = 0.017). We did not observe corrections between glucose homeostasis markers and SP-D genotypes in women with GDM. Furthermore, serum SP-D levels were higher in GDM patients than in matched healthy controls.

**Conclusions:**

This study found the first evidence that an SP-D gene polymorphism (rs721917) was associated with GDM, which may provide the basis for further study on how SP-D plays a regulatory role in GDM.

**Supplementary Information:**

The online version contains supplementary material available at 10.1186/s12884-022-04541-1.

## Background

Gestational diabetes mellitus (GDM), defined as hyperglycemia with onset or first recognition during pregnancy, has recently been recognized and has shown a dramatic increase. According to recent reports, the incidences of GDM in the USA and Europe are 2–10% [1], and in the Chinese population, the incidence is approximately 17.5% [2]. GDM not only increases the risk of a series of current fetal and maternal complications, including dystocia, polyhydramnios, preterm birth, neonatal hypoglycemia, and hyperinsulinemia, but also results in various severe long-term consequences for both the baby and mother, such as predisposition to obesity, metabolic syndrome, and persistent diabetes [3]. Recently, several adipokines have been found to be involved in the pathophysiology of GDM. They have been proven to participate in various metabolic processes, including insulin sensitivity, insulin secretion, appetite control, fat distribution, energy expenditure, inflammation, regulation of adipogenesis, and chemoattraction of immune cells into adipose tissue [4]. Moreover, the alteration of adipokine secretion might contribute to changes in glucose homeostasis in pregnancy, subsequently causing GDM [4].

Surfactant protein D (SP-D, gene name: *SFTPD*) was initially identified to be expressed and secreted in lung alveolar epithelial type II cells [5] and plays a crucial role in protecting the lung from inhaled microorganisms, organic antigens, and toxins by recruiting the innate immune system and consequently regulating inflammatory activities [6]. In addition to the respiratory system, SP-D is also expressed in several other tissues/organs, such as the brain, pancreas, kidney, gut, endothelium, and reproductive system [7–9]. An SP-D polymorphism (rs721917) has been demonstrated to correlate with susceptibility to increased incidence or risk of various disorders, including chronic obstructive pulmonary disease [10], allergic rhinitis [11], asthma [12], and acute kidney injury [13]. Previous studies have revealed the association between low expression of circulating SP-D and increased fat accumulation [14] combined with decreased insulin sensitivity [15], which suggests that SP-D may affect not only specific inflammatory responses but also systemic metabolism [16].

The human SP-D gene located on chromosome 10q22.2-q23.1 contains several single nucleotide polymorphisms (SNPs) [17]. The SP-D single nucleotide polymorphism (SNP) rs721917 (NC_000010.10: g.81706324A > G) is a missense substitution that leads to the replacement of an ancestral methionine by a threonine at position 31 (Met31Thr) [18]. The change from methionine to threonine can cause SP-D oligomeric differences in two common variants.

Some progress has been made in elucidating possible genetic predisposition to GDM through a genome-wide association strategy [19]. According to the GWAS (Genome Wide Association Study), gene defects of coding sequence changes play a role in metabolic disease [20], and compelling data suggest that genetic factors contribute to GDM [21]. A previous study reported that SP-D gene polymorphisms are associated with insulin resistance and type 2 diabetes [22]. However, studies on the genetic susceptibility to GDM are lacking [23]. The research strategies for GDM candidate genes are mainly from genes associated with type 2 diabetes or obesity, which limits the capacity of discovering novel genetic variants of GDM beyond the candidate SNPs of T2D. Therefore, this study aims to investigate whether SP-D polymorphisms are associated with susceptibility to GDM.

## Methods

### Subjects

In this study, we collected data and blood samples from pregnant women diagnosed with GDM and matched healthy pregnant women as controls. Briefly, 147 singleton pregnant women with gestational diabetes aged 24–40 years were admitted to the Department of Obstetrics of Second Hospital Affiliated with Zhejiang University Medical College, Hangzhou, Zhejiang Province, between October 2016 and October 2018, and 97 age-matched healthy singleton pregnancies in the same hospital were recruited as controls. All of the participants recruited were Han Chinese. The diagnosis of GDM was established following the International Association of Diabetes and Pregnancy Study Groups (IADPSG) diagnostic criteria. GDM should be diagnosed at any time in pregnancy if one or more of the listed criteria are met following a 75-g glucose load: fasting PG ≥ 5.1 mmol/l, 1-h PG ≥ 10.0 mmol/l, and 2-h PG of 8.5–11.0 mmol/l. Women diagnosed with DM or prediabetes (impaired fasting glucose or impaired glucose tolerance) before pregnancy were excluded from the study. A total of 97 matched healthy pregnant women were recruited as controls in this study. The study protocol (I2018001239) was approved by the clinical research ethics committee of The Second Affiliated Hospital, School of Medicine, Zhejiang University, Hangzhou, China. Informed consent was signed and obtained from all subjects before initiation of the study. All methods were performed in accordance with the relevant guidelines and regulations.

## Specimen collection

Peripheral blood (2 ml) samples were collected from each GDM patient or healthy control on the day admitted to hospital for delivery. The samples were stored at room temperature for 1 h and then centrifuged at 5000 rpm/min for 5 min to separate white blood cells and serum. The serum and white blood cells were saved at -20 °C for analysis.

## Analysis of the SP-D Thr31Met polymorphism

The SP-D gene polymorphism rs721917 (NC_000010.10: g.81706324A > G) was analyzed using a genomic DNA extraction kit (SolarBio, Beijing, China). The procedure for analyzing the SP-D polymorphism Thr31Met was similar to a previous description, i.e., sequence-specific primer-polymerase chain reaction (PCR-SSP) method [24]. This method provided reproducible results and was as follows: initial denaturation for 1 min at 94 ℃, followed by 5 cycles of 20 s at 94 ℃, 45 s at 65 ℃, and 25 s at 72 ℃, 21 cycles of 25 s at 94 ℃, 50 s at 55 ℃, and 30 s at 72 ℃, 4 cycles of 30 s at 94 ℃, 60 s at 50 ℃, and 120 s at 72 ℃, and a final extension at 72 ℃ for 3 min. Then, the PCR products were separated and identified using 2%-agarose gel electrophoresis. The genotypes of SP-D were determined as described previously [24].

### ELISA of SP-D levels in serum

The serum SP-D protein levels in GDM patients and controls were detected by ELISA (CUSABIO, Shanghai, China) according to the manufacturer’s instructions.

### Sample size

In this study, the distribution differences of three genotypes of the SP-D gene polymorphism rs721917 in pregnant women with gestational diabetes mellitus and healthy pregnant women were investigated. The Chi-square test data analysis method was used to calculate the sample size. In order to explore the difference of moderate effect size, let α = 0.05, Power = 0.95 (β = 0.105), effect size W = 0.3. The sample size calculated by PASS 15 software was 172 cases, and a minimum of 192 cases should be included if 10% shedding rate is considered.

### Statistical analysis

The statistical analysis related to the association between the SP-D gene polymorphism rs721917 and GDM was performed using SPSS software version 20.0 (SPSS Inc., Chicago, IL). Departures from Hardy–Weinberg equilibrium (HWE) were tested to determine whether the genotype and allele frequencies were consistent with the genetic balance. Quantitative data are expressed as mean ± standard deviation and were compared by standard one-way ANOVA or Student's t test. P < 0.05 was considered to be statistically significant. Allele and genotype frequencies were compared by Pearson's two-tailed chi-square test or Fisher’s exact test. The risk of developing GDM under exposure to this SP-D SNP was evaluated using logistic regression to estimate the odds ratio (OR) with a 95% confidence interval, considering the TT genotype as the reference.

## Results

### Baseline characteristics of GDM patients and healthy controls

The clinicopathological characteristics of a total of 147 pregnant women with GDM and 97 healthy pregnant women (controls) in our study are presented in Table 1 (Additional file 2 and 3, GDM and controls). The mean age of GDM patients and the controls was 31.59 ± 3.77 and 29.80 ± 3.85 years, respectively. The mean gestational weeks at delivery of GDM patients and the controls were 38.37 ± 1.19 and 38.47 ± 1.15 weeks, respectively. All of the study populations were delivered at term. Although BMI levels, delivery age, and infant birth age were not different, several markers of glucose homeostasis, including fasting glucose, glucose 1 h post overload, glucose 2 h post overload (mmol/L), and HbA1c (%), differed significantly between GDM patients and healthy controls (*P* < 0.001) (Table 1).

**Table 1:** Baseline characteristics of GDM patients and healthy pregnant controls

	GDM	Controls	*p* value
N	147	97	
Age (yr)	31.59 ± 3.77	29.80 ± 3.85	** < 0.001**
BMI (kg/m^2^)	26.43 ± 2.58	25.97 ± 2.74	0.182
Fasting glucose (mmol/L)	4.70 ± 0.48	4.35 ± 0.52	** < 0.001**
Glucose 1 h post overload (mmol/L)	9.78 ± 1.44	7.48 ± 1.32	** < 0.001**
Glucose 2 h post overload (mmol/L)	8.66 ± 1.30	6.43 ± 1.09	** < 0.001**
HbA1c (%)	4.95 ± 0.35	4.77 ± 0.32	** < 0.001**
Gestational weeks at delivery (w)	38.37 ± 1.19	38.47 ± 1.15	0.520
Infant birth weight (g)	3312.59 ± 440.52	3289.48 ± 379.75	0.673

### Association between SP-D genotype and susceptibility to GDM

The distribution of SP-D Thr31Met genotypes and alleles in this study was consistent with HWE (*P* > 0.05). Compared with healthy controls, the frequency of the SP-D 31Met/Met (T/T) genotype was significantly lower in GDM patients (*P *= 0.033), indicating that homozygous SP-D 31Met/Met (T/T) women are resistant to GDM incidence; the frequency of the SP-D 31Met/Thr (T/C) genotype was significantly higher in GDM patients than in healthy controls (*P* = 0.017), suggesting that heterogeneous SP-D 31Met/Thr (T/C) women are sensitive to GDM incidence (Table 2). However, the frequencies of SP-D 31 Thr (C) and 31 Met (T) alleles were not significantly different in women with GDM and healthy controls (*P* = 0.859) (Table 2). Furthermore, we examined whether there were corrections between SP-D 31Met/Thr genotypes and glucose homeostasis in GDM patients. The results indicated that the markers of glucose homeostasis were not different among the three SP-D genotypes (*P* > 0.05) (Table 3). When the participants with extreme levels of markers of glucose homeostasis in our cohort were analyzed separately, no significant difference was found (*P* > 0.05) (Additional file 1, Supplementary Tables 1-4).

**Table 2:** Surfactant protein D gene polymorphism in GDM patients and healthy controls

Model	GDM		Controls		OR	95% CI	*p* value
	N %		N	%			
Genotype (Met31Thr)							
T/T	17	11.6	21	21.6	0.473	0.235–0.952	**0.033**
C/T	81	55.1	41	42.3	2.440	1.162–5.123	**0.017**
C/C	49	33.3	35	36.1	1.729	0.799–3.745	0.163
Allele							
T	115	39.1	83	42.8	0.859	0.594–1.242	0.419
C	179	60.9	111	57.2

**Table 3:** Baseline serum markers of glucose homeostasis and different genotypes in GDM patients

	TT (17)	TC (81)	CC (49)	*p* value	p* value
Fasting glucose (mmol/L)	4.68 ± 0.47	4.71 ± 0.45	4.69 ± 0.54	0.393	0.448
Glucose 1 h post overload (mmol/L)	9.63 ± 0.86	9.74 ± 1.50	9.88 ± 1.52	0.232	0.522
Glucose 2 h post overload (mmol/L)	8.77 ± 1.19	8.68 ± 1.19	8.58 ± 1.51	0.786	0.646
HbA1c (%)	4.86 ± 0.26	5.01 ± 0.37	4.89 ± 0.36	0.110	0.591
BMI (kg/m2)	26.24 ± 2.01	26.42 ± 2.59	26.52 ± 2.78	0.791	0.706

*P* for the comparison by ANOVA among the different genotypes, and P* for the comparison by ANOVA between TT and CC carriers in the whole cohort

### Serum SP-D levels in GDM patients and healthy controls

The serum SP-D level of healthy controls ranged from 14.63 ng/ml to 36.47 ng/ml (median of 24.68 ng/ml; 25-75^th^ IQR of 19.22–27.63 ng/ml), while the serum SP-D level of GDM patients ranged from 9.44 ng/ml to 60.14 ng/ml (median of 31.06 ng/ml; 25-75^th^ IQR of 14.70–48.65 ng/ml). Compared with healthy controls, the serum SP-D levels in GDM patients were significantly increased (*P* = 0.002).

### Correlation analysis between SP-D genotypes and serum SP-D levels in GDM patients

We further examined whether there exists a correlation between SP-D genotypes and serum SP-D levels. The results showed no difference in serum SP-D levels among the three SP-D genotypes, i.e., Thr31Thr, Met31Thr and Met31Met, in GDM patients (Table 4).

**Table 4:** Basal serum SP-D levels and different genotypes in GDM patients

Genotype	Median (25-75^th^ IQR) baseline serum SP-D (ng/ml)	*p* value
Met31Thr		
T/T	34.88(26.51–54.04)	
C/T	23.86(12.27–43.29)	0.125
C/C	38.41(21.71–56.31)	0.630

*IQR* Interquartile range

## Discussion

The objective of this study was to study the relationship between the SP-D Met31Thr (T/C) polymorphism and susceptibility to GDM in Chinese patients. We found that there exists a significant association of the SP-D polymorphism (rs721917) with GDM. The SP-D (T/T) genotype existed in 11.6% and 21.6% of GDM patients and matched healthy controls, respectively (odds ratio, 0.473; 95% confidence interval, 0.235–0.952; *P* = 0.033), indicating that women with the (T/T) genotype had a lower prevalence of GDM (OR = 0.473). Women with T/C genotypes showed an increased risk of GDM (odds ratio, 2.440; 95% confidence interval, 1.162–5.123; *P* = 0.017). Interestingly, a previous study found that one C-allele carrier showed decreased circulating SP-D and fasting glucose and a lower prevalence of T2D [22]. The levels of glucose homeostasis markers were significantly different in women with GDM compared to matched healthy controls. Furthermore, compared with healthy controls, the serum SP-D levels in GDM patients were significantly elevated (*P* = 0.002), but no difference was observed in the serum SP-D levels between GDM patients with the Thr31Thr genotype and those with the Met31Thr and Met31Met genotypes. To mitigate effects induced by race differences or heterogeneity in regions and living environments, all GDM patients and healthy controls in our study were confined to the Chinese Han nationality residing in similar geographic locations in Zhejiang Province, China. As a result, the frequencies of genotypes at Met31Thr loci in healthy controls were similar to those reported in previous studies conducted in the Chinese population [25], while the distribution of genotypes in the Chinese population differed from that in their Western counterparts [22]. Therefore, we speculated that the SP-D Met31Thr polymorphism might be used as a biomarker to predict Chinese patient susceptibility to GDM.

As an intraexonic polymorphism (rs721917) located in codon 11 in the SP-D N-terminal region, Met31Thr results in changes in the amino acid residues of the SP-D protein. It has been reported that polymorphic variation influences oligomerization, function and circulating concentrations [26]. Leth-Larsen et al. [26] found that the Thr31Thr genotype produces almost exclusively monomers of SP-D, whereas the Met31Met genotype shows multimers such as dodecamers and trimers of subunits. These macromolecule polymers of SP-D may bind to viruses and bacteria, while the monomeric species seem to bind LPS almost exclusively. However, it is unclear how these different oligomeric protein molecules influence other biological functions. Further studies are required in the future.

In the present study, we found that GDM subjects exhibited higher serum SP-D concentrations than healthy controls (*p* = 0.002), particularly among subjects with the Thr31Thr genotype, which might contribute to individual susceptibility. It is worth noting that it is unclear whether the elevated plasma SP-D levels are directly related to gestational diabetes or whether the elevated plasma SP-D levels are due to insulin resistance or inflammatory status in gestational diabetes. However, no difference was observed in the serum SP-D levels between GDM subjects with the Thr31Thr genotype and those with the Met31Thr and Met31Met genotypes. Pueyo et al. [22] found cross-sectional and longitudinal associations of circulating SP-D concentrations with insulin resistance and T2D and described that the Met31Thr SP-D gene SNP rs721917 was associated with insulin resistance and the prevalence of T2D [27]. In this study, GDM partially shares the mechanism of T2D, and the possible influence of the Met31Thr SP-D gene SNP on GDM may arise from changes in the molecular structure and/or functional properties of SP-D protein rather than through changes in its circulating concentrations. Yan et al. [27] verified twenty genes associated with T2D and obesity in a Chinese population and found that only four SNPs were significantly correlated with GDM, and those risk alleles were associated with lipid profiles and glucose levels. Ding et al. [28] genotyped one hundred and twelve susceptibility genetic variants confirmed by genome-wide association studies for T2D from two independent populations and identified only eleven SNPs associated with the risk of GDM. These findings might be explained by two possible reasons. First, mechanisms responsible for incidence or progression vary from gestational diabetes mellitus (GDM) and obesity or its accompanying T2D. In addition, as one of the metabolic diseases, GDM is involved in interactions between genetic and environmental or nutritional factors, which indicates that GDM pathogenesis could be attributed to various elements in addition to genetic polymorphisms. Second, our study was carried out only in the Chinese Han population, which expressed different genetic variants due to race. The sample number was limited. In this study, after extensive adjustment for potential confounders, including maternal BMI, age and parity, we did not observe corrections between glucose homeostasis markers and SP-D genotypes in women patients with GDM, which could be updated in a new study if a larger sample size is applied. Another limitation is that we did not obtain data for maternal serum SP-D information before pregnancy or before GDM development; thus, the causal relationship between serum SP-D level and gestational diabetes remains unclear.

## Conclusions

In summary, we found for the first time that an SP-D gene polymorphism (rs721917) was associated with GDM and that there was a higher serum SP-D level in GDM patients than in matched healthy controls.

## Supplementary Information

**Additional file 1.**

**Additional file 2.**

**Additional file 3.**

## Data Availability

The datasets created and/or analyzed during the current study are available from the corresponding author upon reasonable request.

## Abbreviations

SP-D: Surfactant protein D
GDM: Gestational diabetes mellitus
PCR–RFLP: Cleaved amplification polymorphism sequence-tagged sites
SNP: Single nucleotide polymorphism
CI: Confidence interval
OR: Odd ratio
T2D: Type 2 diabetes
