# Supplementary material for: Association of surfactant protein D gene polymorphism with susceptibility to gestational diabetes mellitus: a case–control study
Source: BMC Pregnancy Childbirth. 2022 Mar 22;22:231. doi: 10.1186/s12884-022-04541-1 (PMC8939171; doi:10.1186/s12884-022-04541-1)
Supplement: Supplementary file 1 — Additional file 1. [file 12884_2022_4541_MOESM1_ESM.docx]

**Supplementary information**

**Association of surfactant protein D gene polymorphism with susceptibility to gestational diabetes mellitus: a case-control study**

Jingwei Xu, Yi Chen, Liangfang Tang, Xinyuan Teng, Lin Feng, Ligui Jin, Guirong Wang, Liquan Wang

**Supplementary Table 1**. Baseline characteristics of a subset of the study population comprising of women with extreme levels of Fasting glucose as considered as belonging to the 1st vs. the 4th quartile of serum fasting glucose concentrations

| Model | Fasting glucose | | Fasting glucose | | P |
| --- | --- | --- | --- | --- | --- |
|  | Quartile I | | Quartile IV | |  |
| N | 37 | | 40 | |  |
| Met31Thr |  |  |  |  |  |
| T/T[N(%)] | 6 | (16.2) | 4 | (10) | 0.507 |
| C/T[N(%)] | 18 | (48.7) | 22 | (55) | 0.577 |
| C/C[N(%)] | 13 | (35.1) | 14 | (35) | 0.990 |
| Age(yr) | 30.95±3.55 | | 32.28±3.95 | | 0.126 |
| BMI(kg/m2) | 25.23±4.83 | | 27.16±3.04 | | 0.038 |

**Supplementary Table 2.** Baseline characteristics of a subset of the study population comprising of women with extreme levels of Glucose1h post overload as considered as belonging to the 1st vs. the 4th quartile of serum glucose1h post overload concentrations

| Model | Glucose1h post overload | | Glucose1hpost overload | | P |
| --- | --- | --- | --- | --- | --- |
|  | Quartile I | | Quartile IV | |  |
| N | 37 | | 38 | |  |
| Met31Thr |  |  |  |  |  |
| T/T[N(%)] | 3 | (8.1) | 1 | (2.6) | 0.358 |
| C/T[N(%)] | 24 | (64.9) | 22 | (57.9) | 0.535 |
| C/C[N(%)] | 10 | (27) | 15 | (39.5) | 0.253 |
| Age (yr) | 30.97±3.08 | | 31.95±3.99 | | 0.241 |
| BMI(kg/m2) | 26.65±2.16 | | 26.32±3.04 | | 0.593 |

**Supplementary Table 3.** Baseline characteristics of a subset of the study population comprising of women with extreme levels of Glucose 2h post overload as considered as belonging to the 1st vs. the 4th quartile of serum glucose2h post overload concentrations

| Model | Glucose2h post overload | | Glucose2hpost overload | | P |
| --- | --- | --- | --- | --- | --- |
|  | Quartile I | | Quartile IV | |  |
| N | 37 | | 38 | |  |
| Met31Thr |  |  |  |  |  |
| T/T[N(%)] | 3 | (8.1) | 4 | (10.5) | 1.000 |
| C/T[N(%)] | 21 | (56.8 | 20 | (52.6) | 0.720 |
| C/C[N(%)] | 13 | (35.1) | 14 | (36.9 | 0.878 |
| Age(yr) | 32.27±4.46 | | 31.37±3.52 | | 0.334 |
| BMI(kg/m2) | 26.77±2.54 | | 26.58±3.21 | | 0.769 |

**Supplementary Table 4.** Baseline characteristics of a subset of the study population comprising of women with extreme levels of HbA1c as considered as belonging to the 1st vs. the 4th quartile of serum HbA1c

| Model | HbA1c | | HbA1c | | P |
| --- | --- | --- | --- | --- | --- |
|  | Quartile I | | Quartile IV | |  |
| N | 40 | | 38 | |  |
| Met31Thr |  |  |  |  |  |
| T/T[N(%)] | 5 | (12.5) | 1 | (2.6) | 0.201 |
| C/T[N(%)] | 19 | (47.5) | 23 | (60.5) | 0.307 |
| C/C[N(%)] | 16 | (40) | 14 | (36.9 | 0.774 |
| Age(yr) | 30.80±3.48 | | 32.47±4.27 | | 0.061 |
| BMI(kg/m2) | 25.94±2.33 | | 27.24±2.65 | | 0.025 |
